# Early life starvation has stronger intra-generational than transgenerational effects on key life-history traits and consumption measures in a sawfly

**DOI:** 10.1371/journal.pone.0226519

**Published:** 2019-12-19

**Authors:** Sarah Catherine Paul, Rocky Putra, Caroline Müller

**Affiliations:** 1 Chemical Ecology, Bielefeld University, Bielefeld, Germany; 2 Hawkesbury Institute for the Environment, Western Sydney University, Penrith, NSW, Australia; Universidade de Sao Paulo, BRAZIL

## Abstract

Resource availability during development shapes not only adult phenotype but also the phenotype of subsequent offspring. When resources are absent and periods of starvation occur in early life, such developmental stress often influences key life-history traits in a way that benefits individuals and their offspring when facing further bouts of starvation. Here we investigated the impacts of different starvation regimes during larval development on life-history traits and measures of consumption in the turnip sawfly, *Athalia rosae* (Hymenoptera: Tenthredinidae). We then assessed whether offspring of starved and non-starved parents differed in their own life-history if reared in conditions that either matched that of their parents or were a mismatch. Early life starvation effects were more pronounced within than across generations in *A*. *rosae*, with negative impacts on adult body mass and increases in developmental time, but no effects on adult longevity in either generation. We found some evidence of higher growth rates in larvae having experienced starvation, although this did not ameliorate the overall negative effect of larval starvation on adult size. However, further work is necessary to disentangle the effects of larval size and instar from those of starvation treatment. Finally, we found weak evidence for transgenerational effects on larval growth, with intra-generational larval starvation experience being more decisive for life-history traits. Our study demonstrates that intra-generational effects of starvation are stronger than transgenerational effects on life-history traits and consumption measures in *A*. *rosae*.

## Introduction

As a major determinant of growth and developmental trajectories, resource abundance in early life plays a crucial role in shaping an individual’s phenotype [1], not least because many of the phenotypic changes that occur in early life persist into adulthood [2,3]. Resource limitation during early life can consequently have long-term detrimental effects on an individual’s phenotype, negatively influencing key life-history traits and ultimately decreasing fitness [4–6]. However, if the resource conditions in early life match those in later life, such alterations in an individual’s phenotype may confer significant fitness advantages [7,8]. Furthermore, these phenotypic changes can be inherited by offspring, resulting in adaptive transgenerational effects if environmental matching persists or the parental condition overall leads to fitness advantages [9–11]. For many organisms, resources are not just scarce but may be absent at some point during development [12]. For example, insects frequently go through ‘boom and bust’ cycles of resource abundance both within and across generations [13]. It is therefore crucial that research into the long-term effects of resource abundance in early life considers not only variation in resource quality or quantity (low to high), but also these periods of acute non-lethal starvation.

Individuals are commonly less resistant to periods of starvation in early life due to the high energetic costs of growth and development [12,14]. However, those individuals that survive such periods of food absence often demonstrate increased resistance to starvation as adults [15,16; but see also 17,18], and have descendants that show similarly elevated levels of starvation resistance, as found, for example, in various insect and nematode species [19,20]. This in turn positively affects the longevity of adults and offspring under certain environmental conditions [15,18,20]. For example, adult honey bees (*Apis mellifera*) starved in early life live longer than individuals raised in an environment of plenty when both are exposed to starvation in adulthood [15].

Enhanced resistance to starvation and consequent knock-on effects on adult longevity, in response to early life starvation, can be caused by a number of factors, including the differential storage and utilisation of energy sources within tissues and a lower metabolic rate [21]. However, such changes in metabolism may only have positive fitness effects, if there is a match between early and late life environments (‘environmental matching’ hypothesis) [22,23], or in the case of transgenerational effects, between parental and offspring early life environments [9,24]. Alternatively, in some cases being born under benign (e.g. non-starvation) conditions and/or to high quality (e.g. non-starved) parents may always be more beneficial for fitness and performance than being born in resource-poor (e.g. starvation) conditions, regardless of an environmental match (‘silver-spoon’ hypothesis) [7,25].

In the face of starvation events in early life and the potential fitness costs of a smaller adult size [26,27], developmental periods are also often extended to enable further growth once resources become available again, so called ‘catch-up growth’ [21,28,29]. Increasing the duration spent in early life phases, however, has associated costs such as increased predation risk [30,31]. These costs may be addressed transgenerationally; for example, in the Glanville fritillary butterfly (*Melitaea cinxia*) larval starvation prolongs developmental time but this prolongation is lower in larvae whose parents also experienced larval starvation, with no impact on pupal mass [32]. An alternative strategy is to increase feeding and developmental rates to enhance consumption efficiency and maximise growth whilst minimising any extension of total development time [33,34]. This so called ‘compensatory’ growth can have associated costs such as decreases in competitive ability against conspecifics [5], mate attractiveness [35], reproductive success [36] and longevity [37]. In comparison to these intra-generational effects much less is known about the transgenerational effects of compensatory growth on offspring phenotype, for example, whether the parents can buffer the long-term costs of catch-up growth in their offspring, when parental and offspring developmental environments match [9]. Such transgenerational effects can be mediated both maternally and paternally [3,38] as well as having sex-specific effects on offspring [39–41].

Here we investigated the effects of starvation in early life, i.e. during larval development, on life-history traits, consumption and growth rates both within and across generations in the turnip sawfly *Athalia rosae* (Hymenoptera: Tenthredinidae). This insect species has a complex life-cycle, where the larvae feed on different species of Brassicaceae, including crop plants on which they can reach pest status [42,43]. In contrast, the adults take up nectar. Multiple eggs are laid on one plant and consequently larvae can go through boom and bust cycles of food availability. Furthermore, several generations are produced each season, thus offspring may experience similar environmental conditions as their parents [44]. In our experimental set-up, larvae of the parental generation (F0) experienced three different regimes of repeated starvation or no starvation. We then used a match-mismatch design to investigate the effects of one of these starvation treatments and the no starvation control on life-history traits and consumption in the progeny (F1). We predicted that in the parental (F0) generation increasing starvation will lead to lower larval survival, prolonged larval development time and a reduced adult body mass, reproductive output and longevity, although larvae may show increased consumption and growth rates after periods of starvation. In the offspring generation (F1), we expected individuals kept under matching conditions to show higher fitness-related trait values than those in mismatching conditions and that larval starvation in the F0 may enhance larval starvation resistance in the F1.

## Materials and methods

**Fig 1:**
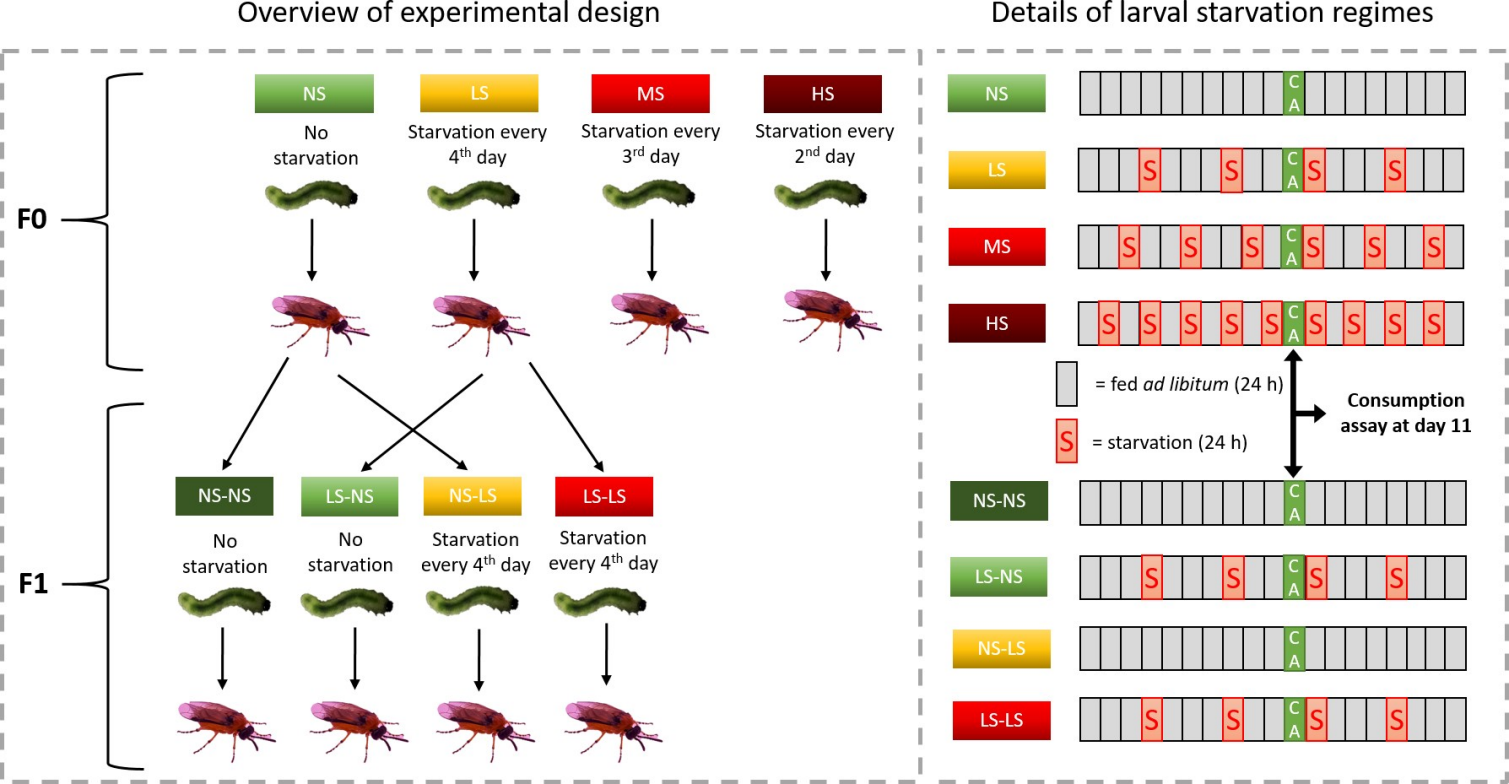
Scheme of the experimental set-up. Larvae of the F0-generation were assigned to four different treatments. F1-individuals from the no starvation (NS) and low starvation (LS) treatment were assorted to either of these two treatments in a match-mismatch design (left). Moreover, details of the starvation regimes and the timing of the consumption assay are depicted (right).

Adults of *A*. *rosae* were collected at a meadow near Bielefeld University, Germany (latitude: 52°2.022'N, longitude: 8°29.718'E; 146 m a.s.l.), and at a number of field margins in the agricultural areas surrounding Bielefeld. Adults were placed together for mating and a culture maintained for approximately 5 generations in cages at room temperature and 16 h: 8 h light:dark circle on potted plants of *Sinapis alba* and *Brassica rapa* (var. *pekinensis*). The plants were grown in a greenhouse (20°C, 16 h: 8 h light:dark, 70% r.h.). For the experiment, 45 female and 20 male adults (parent generation) were set up in a cage with flowering *S*. *alba* plants, which is the preferred plant for oviposition (Müller, unpublished). One hundred newly emerged larvae (F0) were placed individually in Petri dishes (5.5 cm diameter) lined with filter paper and provided with leaf discs cut from middle-aged leaves of 6–8 weeks old *B*. *rapa* plants, standardising leaf age, and thus plant quality, as much as possible. In contrast to *S*. *alba*, cut leaf discs of *B*. *rapa* stay in a good condition at least for 24–48 h and therefore are more suitable for rearing and consumption experiments. These larvae were kept in a climate chamber at a cycle of 20°C for 16 h at day:16°C for 8 h at night and 70% r.h. To test the effects of different starvation regimes on life-history traits, larvae were randomly assigned to one of four treatment groups (25 larvae per group). The no starvation (NS) group received food *ad libitum*. Larvae of the low starvation (LS) group were provided with leaf discs in a rotating cycle of three days *ad libitum* supply and one day (24 h) of starvation. The moderate starvation (MS) group received *B*. *rapa* leaf discs in a rotating cycle of two days *ad libitum* supply and one day (24 h) of starvation. Larvae of the high starvation (HS) group were starved every other day for 24 h (i.e. alternatingly 1 d food, 1 d starvation etc, Fig 1). These treatments were applied until the larvae entered the eonymph stage (non-feeding, final larval instar).

On reaching the eonymph stage, eonymphs were placed in plastic cups containing ~30 g soil for pupation and cups were covered with a gauze lid. The cups were checked daily for emerging adults to measure pupal development time and survival. Freshly emerged adults were weighed on a microbalance (ME36S, accuracy 0.001 mg; Sartorius, Göttingen, Germany), kept individually in Petri dishes, provided with a mixture of honey and water (1:10) and adults checked daily for their status (alive/dead) to measure adult longevity. Within each treatment group, pairs of one female and one male were set up for mating, which usually occurs within less than half an hour. Because fewer males than females emerged in the F0 generation, some males were mated with more than one female. Males were kept afterwards in Petri dishes until death. Females were placed in plastic cups (0.3 L), provided with the honey-water mix and offered leaves of *S*. *alba* for oviposition until death. The total number of hatching larvae (F1) produced by each female (F0) was counted.

To test the combined effects of parental and offspring larval starvation treatment on offspring life-history traits, F1 larvae hatching from eggs of F0 females in the NS and LS treatment groups were used (as the number of surviving larvae was highest in these treatment groups). F1 larvae were then assigned to a starvation treatment in a match-mismatch design [45] where their rearing environment either matched that of their parents (NS-NS, LS-LS) or did not (LS-NS, NS-LS), resulting in four different larval rearing treatments (Fig 1). F1 larvae derived from F0 parents of the NS group received either food *ad libitum* (NS-NS; n = 12) or were reared on *B*. *rapa* leaf discs in a rotating cycle of three days *ad libitum* supply and one day of starvation (NS-LS; n = 12). F1 larvae derived from F0 parents of the LS group received either food *ad libitum* (LS-NS; n = 19) or reared on *B*. *rapa* leaf discs in a rotating cycle of three days *ad libitum* supply and one day of starvation (LS-LS; n = 21). Larval survival, larval and pupal development time, adult body mass and longevity were recorded for the F1 individuals as done for the F0 individuals.

Consumption rates for larvae from both the F0 and F1 generation were measured when larvae were 11 days post hatching. Each larva was weighed (= initial body mass) and supplied with a defined number of *B*. *rapa* leaf discs (surface area of 219.28 mm^2^, corresponding to a fresh mass of 64.88 mg, average of 20 weighed leaf discs). After 24 hours, larvae were weighed again (= final body mass), and the leaf disc remains were scanned (Samsung SAMS M3375FD, resolution 640 x 480). The remaining leaf area was calculated from images in Image J, using the thresholding tool to ensure accurate area selection. The remaining area was then used to calculate the total mass of consumed leaf (mg).

### Statistical analysis

Data were analysed using R version 3.4.4 (R Core Team, 2018). The alpha level was set at 0.05 for all tests. Data of the F0 generation were analysed using general linear (lm) and generalised linear models (glm) (package:MASS; see below for full model details) and data of the F1 generation using liner mixed effects models (package:lme4; see below for full description of each model). Model residuals were checked for normality and variance homogeneity. Stepwise backwards deletion using Chi-square likelihood ratio tests (package:MASS) was employed to reach the minimum adequate model [46]. Posthoc analyses were carried out using the package ‘multcomp’ (package:multcomp, [47]).

Parental (F0) generation: The influence of the four different larval starvation treatments on the total number of surviving larvae in the F0 generation was assessed using a glm with a binomial distribution and logit link. Due to the low number of surviving larvae the HS treatment was dropped from further analyses of life-history traits and consumption measures in the F0 generation (see Results section for further details). The effects of the three remaining starvation treatment levels (NS, LS and MS), sex and their interaction on larval development time were tested using a glm with a Poisson distribution and log link. The effects of larval starvation treatment and sex on adult survival were assessed *via* a survival analysis (package: Survival, [48]). The survfit function was used to produce Kaplan-Meier survival curves and the difference between these curves was tested using a log-rank test with the survdiff function. A linear model was used to establish the effects of larval starvation treatment (NS, LS and MS), sex and their interaction on adult body mass (mg). The influence of starvation treatment (NS, LS and MS), female body mass (mg), and their interaction on the total number of hatched larvae per female was assessed using a negative binomial glm to account for overdispersion in the model.

We estimated relative growth rate (RGR), relative consumption rate (RCR) and food conversion efficiency (ECI) of larvae using general linear models [49], to avoid the pitfalls associated with the use of ratios when calculating nutritional and growth parameters [50,51]. Furthermore, due to reduced feeding time whilst undergoing ecdysis, those larvae that moulted to the next instar during the consumption assays were excluded from the analyses. The RGR was estimated *via* analysis of the change in larval body mass [final mass (mg)—initial mass (mg)] as the response variable and initial larval body mass, larval starvation treatment, sex, and two two-way interactions between initial larval body mass and larval starvation treatment or sex as the predictors. The RCR was estimated *via* the analysis of the log of total fresh mass of consumed leaf material (mg) as the response variable and the same predictors as for the RGR analysis. The ECI was estimated *via* the analysis of the change in larval body mass as the response variable and the total fresh mass of consumed leaf material, larval starvation treatment, sex, and two two-way interactions between mass of consumed leaf material and larval starvation treatment or sex as the predictors.

Offspring (F1) generation: Due to the low number of females (n = 6) compared to males (n = 64) reaching adult eclosion in the F1 generation, the full analysis was carried out only on males (NS-NS = 11, NS-LS = 10, LS-NS = 17, LS-LS = 17). The influence of starvation treatment in both the parental and offspring generation on larval developmental time was assessed using a GLM (family = Poisson, link = log; package: BASE), where larval development time (days) was the response variable, and parental (F0) larval starvation treatment (NS or LS), offspring (F1) starvation treatment (NS or LS) and their interaction were the predictors. A survival analysis (package: Survival, [48]) was used to establish the effect of parental (NS or LS), offspring larval starvation treatment (NS or LS), and their interaction on adult survival. The survfit function was used to produce Kaplan-Meier survival curves and the difference between these curves was tested using a log-rank test with the survdiff function. The effects of parental (NS or LS) and offspring larval starvation treatment (NS or LS) and their interaction on offspring adult body mass (mg) were assessed using a linear model (package: BASE). As with the parental (F0) generation, we excluded larvae that moulted during consumption assays from the analysis of RGR, RCR and ECI of larvae. The RGR was estimated *via* the analysis of the change in larval body mass [final mass (mg)—initial mass (mg)] as the response variable and initial larval body mass, parental (F0) larval starvation treatment (NS or LS), offspring (F1) starvation treatment (NS or LS) and their interactions (three-way interaction) as predictors. The RCR was estimated *via* the analysis of the log of total fresh mass of consumed leaf material (mg) as the response variable and initial larval body mass, parental (F0) larval starvation treatment (NS or LS), offspring (F1) starvation treatment (NS or LS) and their interactions (three-way interaction) as predictors. The ECI was estimated *via* the analysis of the change in larval body mass as the response variable and the total fresh mass of consumed leaf material, parental (F0) larval starvation treatment (NS or LS), offspring (F1) starvation treatment (NS or LS) and their interactions (three-way interaction) as predictors.

## Results

**Fig 2:**
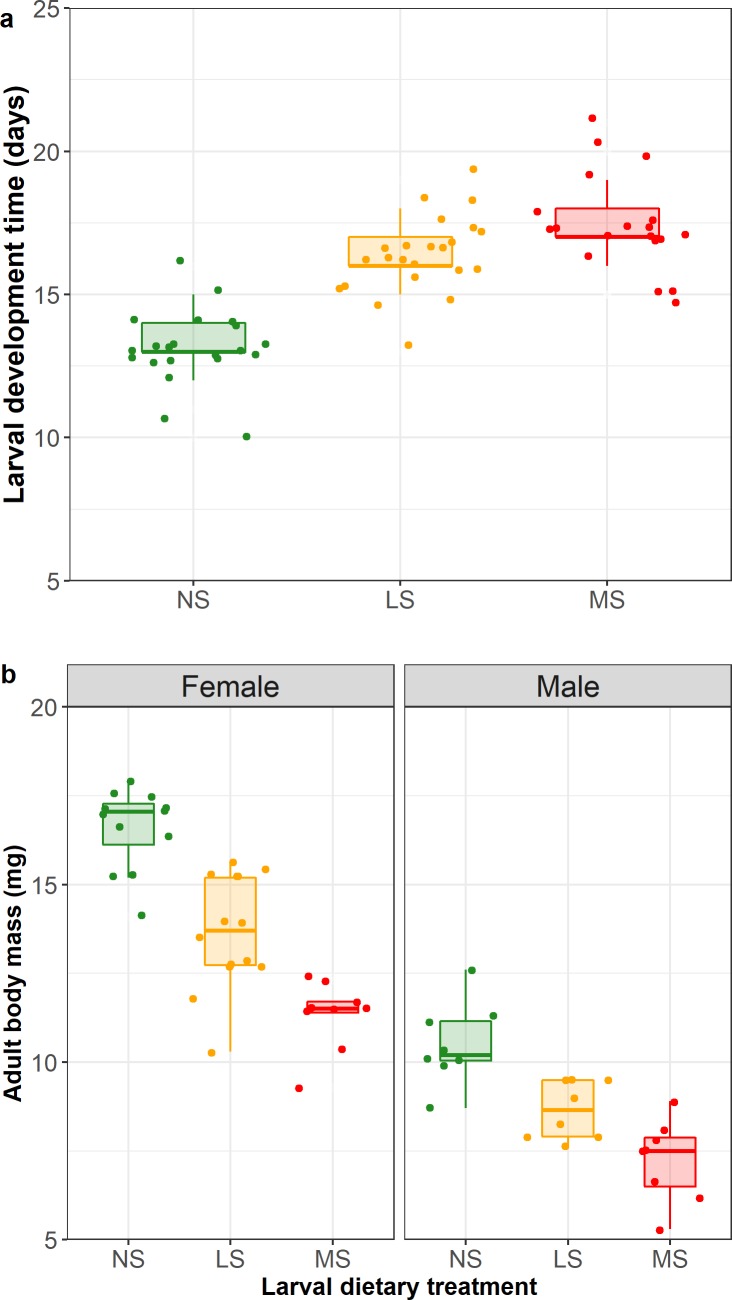
Effect of larval starvation treatment on larval development time (a) and effects of larval starvation treatment and sex on adult body mass (b) of the parental generation (F0) of *Athalia rosae*. Box plots show the medians and 25th and 75th percentiles; the whiskers indicate the values within 1.5 times the interquartile range and are overlaid with raw data points.

**Fig 3:**
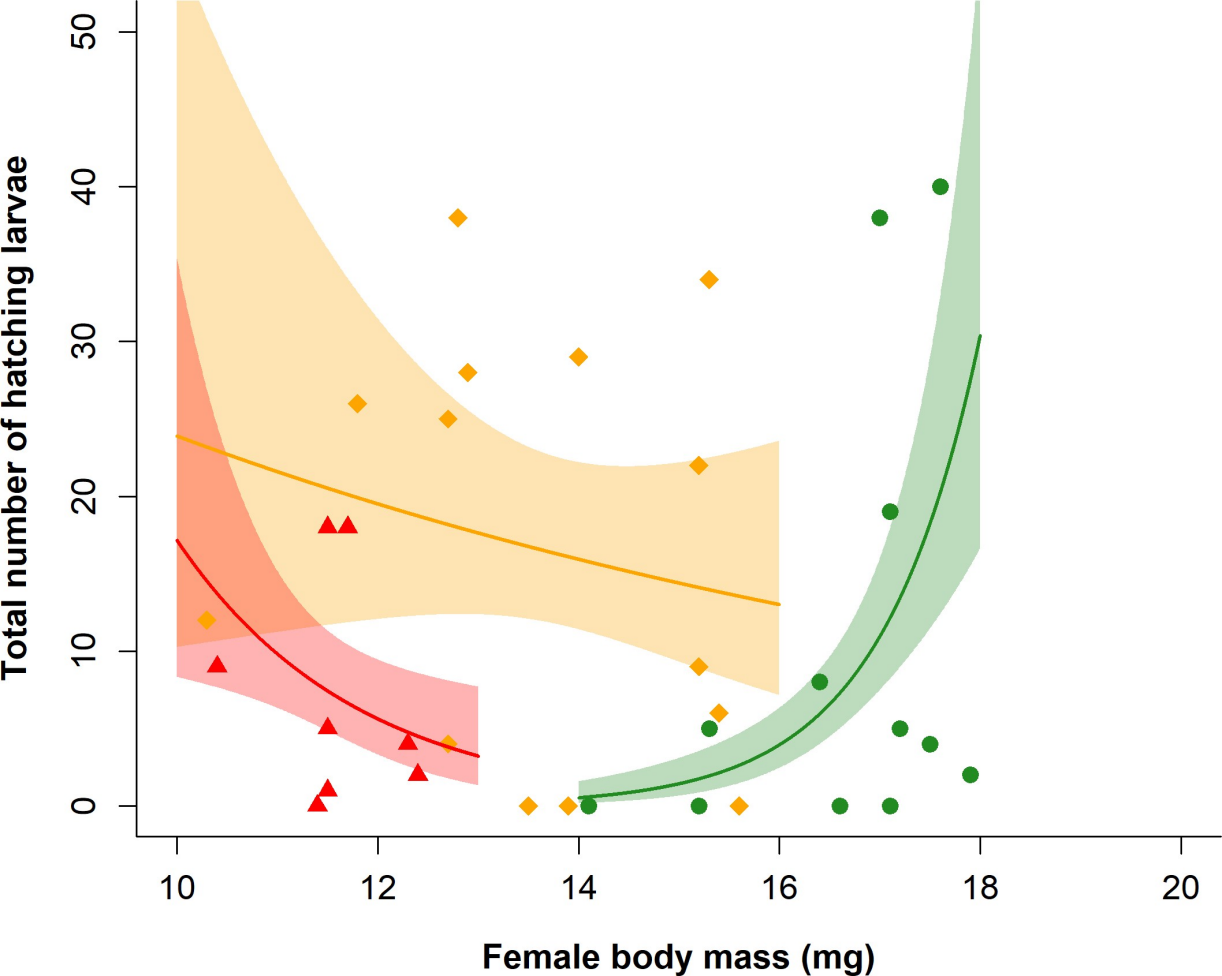
Effect of larval food treatment [green circles–no starvation (NS), orange diamonds–low starvation (LS), red triangles–moderate starvation (MS)] on the relationship between total number of larvae hatching from eggs laid by *Athalia rosae* F0 females and female body mass (mg). Lines represent model predictions with associated SE plotted as polygons around each line and overlaid with raw data points.

In the F0 generation, larvae of the high starvation treatment (HS) had a significantly lower larval survival than larvae of the three other starvation treatment levels (total number of larvae reaching pupation: HS = 8%, MS = 76%, LS = 92%, NS = 84%; X^2^_3,96_ = 52.08, p < 0.001). Larval development time was significantly longer when larvae had periods of starvation (LS, MS) than when they were fed *ad libitum* (NS) (X^2^_2,60_ = 13.17, p = 0.001; pairwise comparisons: NS *vs*. MS z = -3.42, p = 0.002; NS *vs*. LS z = -2.79, p = 0.015; LS *vs*. MS z = -0.77, p = 0.72; Fig 2A). However, there was no significant interactive effect of sex with treatment (X^2^_2,57_ = 0.50, p = 0.77) or sex alone on larval developmental time (X^2^_1,59_ = 0.39, p = 0.53). In contrast, sex influenced adult longevity, with females living significantly longer than males (log-rank test X^2^ = 7.9_1_, p = 0.005), independent of larval starvation treatment (NS treatment * sex interaction log-rank test X^2^ = 9.8_5_, p = 0.08 and main treatment effect log-rank test X^2^ = 0.3_2_, p = 0.9). Adult females were heavier than adult males and adult body mass decreased with increasing bouts of starvation (treatment: F_2,55_ = 57.43, p < 0.001; sex: F_1,55_ = 234.44, p < 0.001; Fig 2B), although the interaction between treatment and sex was not significant (treatment * sex: F_2,53_ = 3.03, p = 0.057). The total number of hatched larvae per female varied with female body mass in dependence of the larval starvation treatment (treatment * maternal body mass: X^2^_2,29_ = 6.98, p = 0.03). Females of the MS or LS treatment showed a decreasing number of hatched larvae with increasing female body mass, whereas females of the NS treatment showed the reverse (Fig 3).

**Fig 4:**
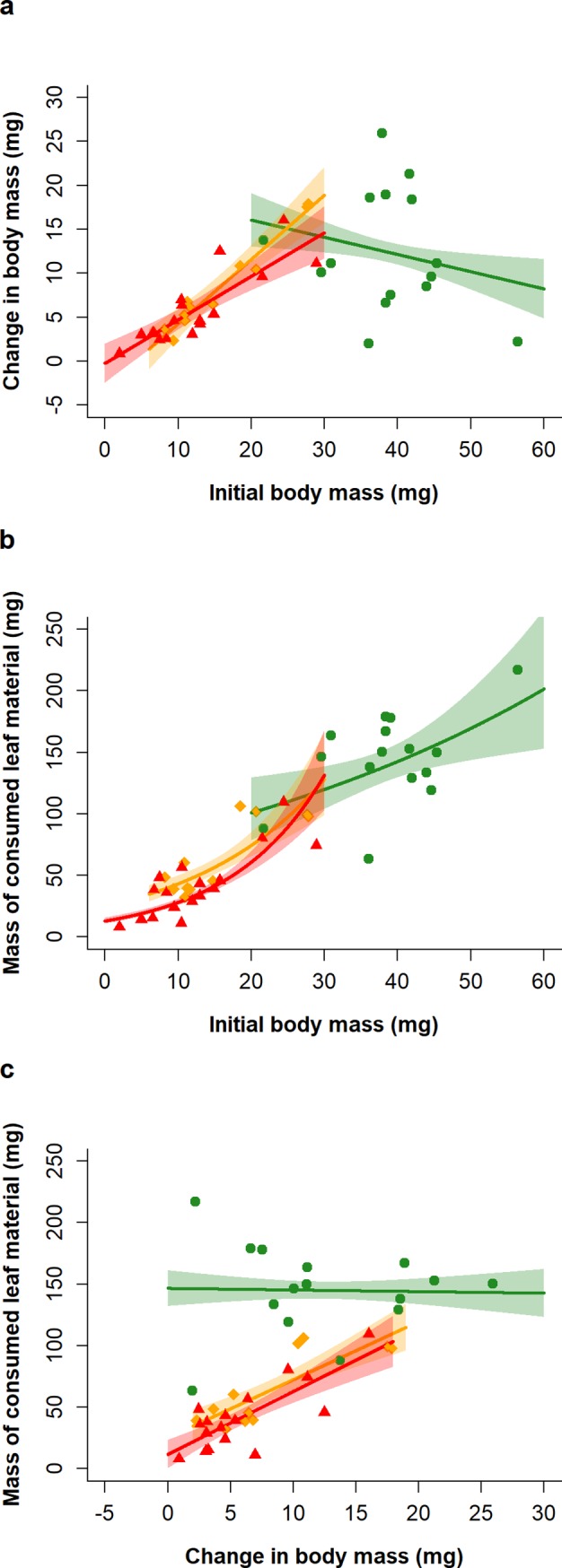
Results of consumption assays of larvae of *Athalia rosae* in the parental (F0) generation; relative growth rate (RGR; a), i.e. the relationship between their change in body mass during the consumption assay and their initial body mass at the start of the assay; relative consumption rate (RCR; b), i.e. the relationship between the amount of food consumed by larvae and their initial body mass; efficiency of food conversion (ECI; c), estimated *via* the analysis of the change in larval body mass and the total leaf consumption over the course of the consumption assay. Larvae experienced either no starvation (green circles), low starvation (orange diamonds) or moderate starvation (red triangles). Lines represent model predictions with associated SE plotted as polygons around each line and overlaid with raw data points.

The relative growth rate (RGR) differed significantly between F0 larvae of the different starvation treatments (larval initial body mass * treatment: F_2,33_ = 15.04, p < 0.001; Fig 4A). There was a positive relationship between initial body mass and changing body mass in larvae of both starvation treatments (LS, MS), but this relationship was negative for larvae of the NS treatment (Fig 4A). The relationship between initial body mass and changing body mass was positive for both sexes, although the initial mass and overall mass change was smaller for males than for females (sex * treatment: F_1,33_ = 5.85, p = 0.021). Across all treatments there was a significant positive relationship between the amount of leaf material consumed by larvae and their initial body mass, i.e. the RCR (Fig 4B). This relationship also differed significantly between larvae kept at different larval starvation treatments (larval initial body mass * treatment: F_2,37_ = 5.76, p = 0.007), with increases in the initial body mass of larvae that had previously been starved (LS, MS) resulting in larger increases in the amount of consumed leaf material than equivalent increases in initial mass in larvae of the NS treatment (Fig 4B). There was no significant effect of sex on either leaf consumption (F_1,34_ = 0.10, p = 0.76) or on the relationship between leaf consumption and initial larval body mass (F_1,33_ = 0.51, p = 0.48).

The ECI differed significantly between F0 larvae of the different treatments (change in body mass * treatment: F_2,37_ = 5.39, p = 0.009). In both starvation treatments (LS, MS) larvae that had a higher increase in body mass consumed more food over the course of the consumption assay, while in larvae of the NS treatment there was no apparent relationship between consumption and body mass change (Fig 4C). There was no significant effect of sex on either leaf consumption (F_1,34_ = 1.57, p = 0.22) or on the relationship between leaf consumption and larval change in body mass (F_1,33_ = 0.31, p = 0.58).

**Fig 5:**
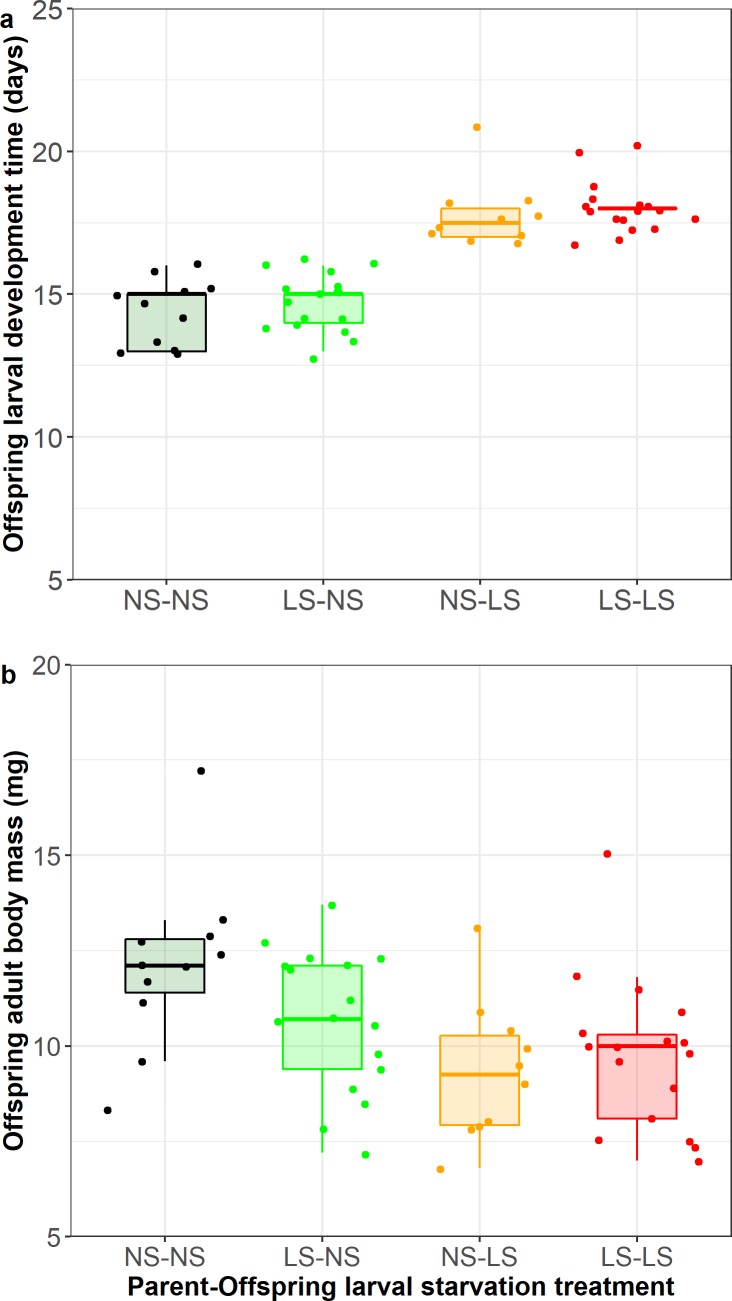
Development time (a) and male adult body mass (b) of *Athalia rosae* larvae in offspring (F1) generation split by parental and offspring larval starvation treatment; larvae experienced either no starvation (NS) or low starvation (LS) in a match-mismatch design. Box plots show the medians and 25th and 75th percentiles; the whiskers indicate the values within 1.5 times the interquartile range and are overlaid with raw data points.

In the F1 generation, there was a significant effect of offspring larval diet on larval developmental time, with larvae that were fed *ad libitum* without any starvation having shorter development times than those that had periods of starvation (X^2^_1,53_ = 9.74, p = 0.002; Fig 5A). Starvation treatment of the parental larvae had no effect on developmental time of the F1 larvae (parental larval diet * offspring larval diet: X^2^_1,51_ = 0.07, p = 0.79). There was no significant effect of either offspring larval starvation treatment (log-rank test X^2^ = 0.6_1_, p = 0.5), parental larval starvation treatment (log-rank test X^2^ = 1.1_1_, p = 0.3) or their interaction (log-rank test X^2^ = 1.8_3_, p = 0.6) on offspring adult longevity. Male adult body mass differed depending on starvation treatment; F1 adults were heavier when reared as larvae without starvation (NS) (F_1,53_ = 9.51, p = 0.003; Fig 5B), and there was a non-significant trend for this difference to be higher when the parental larvae also experienced no starvation (parental larval diet * offspring larval diet: F_1,51_ = 2.77, p = 0.10; Fig 5B).

**Fig 6:**
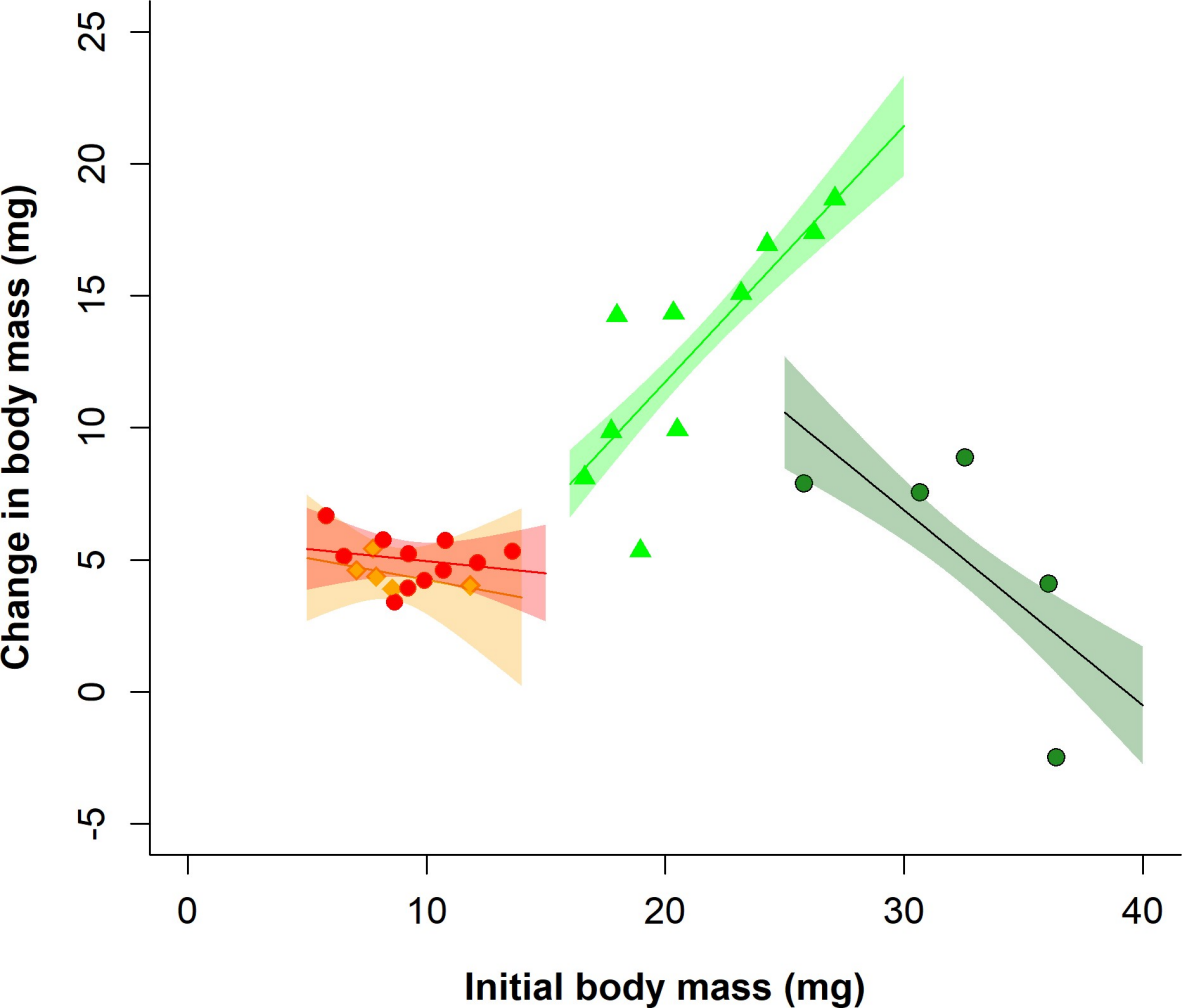
Relationship between the change in mass during the consumption assay and the mass at the start of the assay, or relative growth rate (RGR), of *Athalia rosae* larvae in the offspring (F1) generation. Data is split by parental and offspring larval starvation treatments, combinations of no starvation (NS) and low starvation (LS); LS-LS = red circles, NS-LS = orange diamonds, LS-NS = light green triangles, NS-NS = dark green circles. Lines and polygons are model predictions and associated standard errors (SE), overlaid with raw data points.

There was a significant difference in the relationship between the initial larval body mass and the change in larval body mass (RGR) between F1 larvae of different treatments in the consumption assay (larval initial body mass * parental larval diet * offspring larval diet: F_1,23_ = 4.76, P = 0.04; Fig 6). F1 larvae which were either subject to a starvation treatment themselves (LS-LS and NS-LS) or whose parents had been starved as larvae (LS-NS) showed a positive relationship between larval initial body mass and change in body mass, whereas larvae of the NS-NS treatment showed a negative relationship (Fig 6). However, neither parental larval starvation treatment (larval initial body mass * parental larval diet: F_1,25_ = 2.72, P = 0.11), offspring larval starvation treatment (larval initial body mass * offspring larval diet: F_1,25_ = 0.67, P = 0.42) nor their interaction (larval initial body mass * parental larval diet * offspring larval diet: F_1,23_ = 0.56, P = 0.46) influenced the relationship between larval leaf consumption and larval initial body mass, i.e. RCR. Likewise, these two treatments and their interaction did not influence the relationship between larval leaf consumption and the change in larval body mass, i.e. ECI, in the consumption assay (larval change in body mass * parental larval diet * offspring larval diet: F_1,23_ = 1.29, P = 0.27; larval change in body mass * parental larval diet: F_1,25_ = 0.61, P = 0.44; larval change in body mass * offspring larval diet: F_1,25_ = 0.58, P = 0.45).

## Discussion

Here we aimed to assess the influence of early life starvation on life-history traits and consumption measures both within and across generations in the turnip sawfly *A*. *rosae*. Whilst our results demonstrate the negative impacts of starvation in early life on survival, development time and adult body mass, the effects of larval starvation on consumption and growth were more complex. Furthermore, although there was some weak evidence for transgenerational effects of parental diet on offspring, overall the effects of early life diet on key life-history traits were more pronounced within than across generations in *A*. *rosae*.

### F0 generation—Early life effects

In the parental (F0) generation, periods of starvation during the larval phase prolonged larval developmental time. Increases in developmental time are often linked to starved individuals ‘catching up’ the overall growth achieved by non-starved individuals [33]. However, this was not the case for starved *A*. *rosae* larvae, which despite their prolonged developmental period were smaller as adults than those individuals raised without starvation. In fact, a prolongation of developmental time in response to early life dietary manipulation does not always result in a complete catch-up in terms of adult size [52]. Adult mass also decreased with increasing frequency of larval starvation bouts, although the developmental time of individuals in these treatments (LS *vs*. MS) did not differ. These results suggest that there might be a limit to the prolongation of developmental periods in *A*. *rosae* in the face of harsh early life conditions, resulting in a trade-off with adult body mass. Several factors might select against extended larval development including increased risk of predation and parasitism as well as seasonal pressures such as peak adult food abundance, temperature or mate availability [30,31,53–55]. Alternatively, for holometabolous species such as *A*. *rosae* where only adults have high mobility, it may be advantageous to complete development at a smaller size to enable dispersal, whilst differentially allocating available resources during metamorphosis to improve dispersal abilities [26,56,57]. For example, in the butterfly *Bicyclus anynana* resource constraints in early life lead to a lower adult body mass, with these smaller adults having a higher thorax ratio (thorax dry mass/total dry mass) resulting in a better flight performance compared to larger adults [58].

In terms of treatment effects on the growth rate of *A*. *rosae*, those individuals that were starved appeared to have a higher growth rate than those individuals that did not experience starvation. Likewise, previously starved larvae also showed more efficient consumption, despite all individuals consuming a larger amount of leaf material with increasing larval size. This might indicate ‘compensatory growth’, i.e. an increased growth rate, in starved larvae once food is available [33], however, one should be cautious of this interpretation [34]. The assays were carried out 11 days after hatching and consequently the larvae reared under no starvation conditions were much larger than those in both of the starvation treatments and were closer to the final larval ecdysis. Growth rate commonly decreases with increasing size due to the energetic demands of somatic maintenance [59] and therefore may also vary across larval instars. Thus, further work comparing the consumption and growth of larvae from different starvation treatments at more comparable sizes and instars is needed to conclusively assess whether *A*. *rosae* employs compensatory growth when recovering from larval starvation.

Starvation bouts during development had no effect on adult longevity (F0 generation). However, empirical work on the relationship between early life starvation and adult longevity provides a mixed bag of results, spanning the spectrum from positive to negative effects on adult lifespan [60–62]. Arguably, such variation could partly be attributed to a conflation of the different ways in which diet is varied across different studies, either by restriction of key nutrients (diet quality) or general caloric restriction (quantity) [61], resulting in markedly different physiological effects. Severe stress in early life such as that caused by starvation can carry with it costs associated with somatic damage, e.g., increased telomere attrition [63–66]. The predictions of environmental matching suggest that despite such effects, adult lifespan is maximised when resource conditions in early and late life match, as is the case in *A*. *mellifera* [15]. In other cases the negative somatic effects of early life resource constraints or other early life stressors are often more pronounced under stressful adult conditions and increase with the intensity of the stress, e.g. longer or more frequent bouts of starvation [67]. This may therefore explain why we found no difference in longevity of adult *A*. *rosae* based on early life experience, as the environment for all adults was benign, with a constant food supply.

Starvation also altered the relationship between female body mass and reproductive output in the parental generation, with a positive relationship in individuals experiencing no starvation and a negative relationship in individuals starved during development. Starvation and resource restriction in early life frequently reduce female fecundity [20,29,68], in part due to enhanced resource allocation to growth and survival opposed to the organs involved in reproduction [15,69]. Early life starvation experienced by mothers can also potentially act as a cue indicating a poor future offspring environment [9]. Maximising offspring size in the face of such cues results in fitness benefits for offspring [70], especially in the face of adverse environmental conditions such as starvation [71,72]. However, responding to these cues can result in a trade-off between offspring number and size [73], as, for example, found in females of *B*. *anynana* that were starved in early life [58]. This trade-off may have contributed to the negative relationship observed in *A*. *rosae* between maternal size and offspring number in the starvation treatments, although future measurements of offspring size would be necessary to test this assumption.

### F1 generation—Early life and transgenerational effects

The starvation regime of larvae in the offspring generation (F1) had a more pronounced effect on offspring life-history traits of *A*. *rosae* than the parental starvation treatment. Neither parental nor offspring larval starvation treatments influenced offspring adult lifespan, but offspring larval starvation increased developmental time and decreased male adult body mass independent of parental conditions. In contrast, in the grasshopper *Chorthippus biguttulus* high quality diet of the parental generation had positive effects on the offspring developmental time and body mass, when diet quality was kept high or low over the entire development of each generation [74]. In *Drosophila melanogaster*, offspring of mothers raised as larvae under poor environmental conditions had a larger size and shorter development time than the offspring of mothers that developed in a resource-abundant environment [75]. Thus, intra- *versus* transgenerational responses may be highly species- and dietary treatment-specific. Furthermore, both offspring and adult larval starvation treatments interactively affected offspring larval growth rate in *A*. *rosae*. The contrasting growth rates in particular between larvae of the LS-NS and the NS-NS treatment are most striking, as they indicate that the experience of poor larval conditions in the parental generation enhances the growth rate in offspring. However, as discussed above for the F0 generation, the different growth trajectories are probably mostly related to the body size and ontogeny of the larvae, with increasing growth rates up to a certain larval size and decreases once larvae approach pupation.

The environmental matching hypothesis predicts fitness benefits when the environment matches between parents and offspring [9,24]. Thus, one might expect starved individuals from parents that themselves were starved to perform better than progeny from parents not starved as larvae (i.e. LS-LS > NS-LS). In both a butterfly and a springtail species, for example, offspring of individuals that had experienced starvation or low food abundance had a shorter development time and a higher adult body mass when exposed to such conditions themselves during development than those individuals from parents reared under benign conditions, although for springtails this was true only for female offspring [41,76]. Alternatively, the silver-spoon hypothesis predicts that positive effects of high quality parents benefit not only those offspring in good environments, but also those in poor environments [7,25], thus leading to the opposite effect (i.e., here expecting LS-LS < HS-LS). Support for the silver-spoon effect has been found in insects and birds [74,77]. However, no robust support for either of these effects (environmental matching or silver-spoon) was found in the current study in offspring of *A*. *rosae*, with starved individuals from parents that themselves were starved or not starved as larvae (LS-LS and NS-LS) having similar growth trajectories. One possible explanation is that the benefits of having high quality parents may mask the benefits of environmental matching, when offspring are developing in a poor environment. These types of transgenerational effects can be difficult to disentangle with a match-mismatch design, because the effects of both parental and offspring environments may not necessarily act in an additive manner [78]. Overall, there appears to be only weak evidence for transgenerational effects of parental diet experience on offspring in *A*. *rosae*, with much larger environmental effects of offspring diet.

### Conclusion

The effects of early life diet experience on key life-history traits were stronger within than across generations in *A*. *rosae*. Early life starvation negatively influenced adult body mass and increased individual developmental time, with growth trajectories in this experiment likely predominantly determined by current body size and ontogeny. Further work is necessary to disentangle the effects of larval size and instar from that of starvation treatment when studying consumption and growth rates in this species. Future studies may also consider the effects of single acute starvation bouts, simulating alternative field conditions where high numbers of later larval instars can decimate single host plants before finishing development. As the physiological effects of starvation are known to differ between different rates and duration of starvation bouts, there might also be differences in the intra-generational and transgenerational life-history responses of individuals to these different types of starvation.

## Supporting information

S1 Table Complete raw data set of the experiments. (XLSX)
